# Peer Actors and Theater Techniques Play Pivotal Roles in Improving Social Play and Anxiety for Children With Autism

**DOI:** 10.3389/fpsyg.2020.00908

**Published:** 2020-05-27

**Authors:** Sara Ioannou, Alexandra P. Key, Rachael A. Muscatello, Mark Klemencic, Blythe A. Corbett

**Affiliations:** ^1^Department of Psychiatry and Behavioral Sciences, Vanderbilt University Medical Center, Nashville, TN, United States; ^2^Vanderbilt Kennedy Center, Vanderbilt University Medical Center, Nashville, TN, United States; ^3^Department of Hearing and Speech Sciences, Vanderbilt University Medical Center, Nashville, TN, United States; ^4^Vanderbilt Brain Institute, Vanderbilt University, Nashville, TN, United States; ^5^Department of Psychology, Vanderbilt University, Nashville, TN, United States

**Keywords:** autism, theater, play, anxiety, peers

## Abstract

Children with autism spectrum disorder (ASD) have significant difficulty in social functioning to include engaging in natural play with peers. Many children with ASD exhibit significantly less interactive play and more physiological stress during benign social encounters with same-age peers on a playground. Theatrical role-playing and performance with expert role models may provide a unique opportunity for children with ASD to learn to engage with other children in a safe, supportive environment. SENSE Theatre^®^ is a peer-mediated, theater-based program aimed at improving social competence in youth with ASD. Previous studies have shown significant improvements in social and communication skills following SENSE Theatre^®^ intervention. The current project examined play with novel peers and self-reported anxiety before and after participation in SENSE Theatre^®^. Participants included 77 children between 8 and 16 years with high-functioning (IQ ≥ 70) ASD. The combined sample of three cohorts was randomized to the experimental (EXP, *N* = 44) or waitlist control (WLC, *N* = 33) group. Participants in the EXP group received 40 h (10, 4-h sessions) of SENSE Theatre^®^. The Peer Interaction Paradigm (PIP), an ecologically valid measure of natural play, was administered before and after the intervention. Group Play and Self Play on the playground equipment during solicited (T4) and unsolicited (T1) play were used in the current study. The State Trait Anxiety Scale for Children (STAIC; Spielberger et al., 1983) was used to measure self-reported current and persistent anxiety, respectively. Following treatment, children in the EXP group engaged in significantly more Group Play with novel peers [*F*(2,73) = 7.78, *p* = 0.007] and much less Self Play [*F*(2,73) = 6.70, *p* = 0.01] during solicited play compared to the WLC group. Regression analysis revealed that pretreatment play and group status were significant predictors of solicited Group Play. Children in the EXP group reported significantly less Trait anxiety following intervention [*F*(2,71) = 6.87, *p* = 0.01]; however, State anxiety was comparable. Results corroborate previous findings of significant changes in social and play behavior in children with ASD following the peer-mediated, theater-based intervention. Acting and theatrical performance with supportive role models facilitates social engagement in everyday settings with novel peers and reductions in self-reported anxiety.

## Introduction

Play is notoriously difficult to define but is generally thought to incorporate flexibility, non-literality, pleasure, spontaneity, and active engagement (Krasnor and Pepler, 1980; Garvey, 1999). Play has long been considered a vital component of a child’s development (Vygotsky, 1978) and has a key role in the acquisition of multiple cognitive, socioemotional, and communicative skills. For example, play cultivates social referencing, role-taking, and symbolism (Lillard et al., 2011). Various social communication skills such as conflict resolution, negotiation (Sawyer, 1997; Frost, 1998; Ginsburg, 2007), and perspective taking (Burns and Brainerd, 1979; Jordan, 2003) are also built through play.

Indeed, communication and language are fundamentally connected to play particularly through the use of symbols (McCarthren et al., 1996). As language involves using words to symbolize what one is aiming to convey, play involves using pretense to symbolize meaning (Mccathren et al., 1999). For instance, Laakso et al. (1999) found that children’s symbolic play abilities predicted later language comprehension skills. In this context, the definition by Leslie (1988) is adopted in which pretend play can have three basic forms or properties to include (a) object substitution, (b) attribution of false properties, or (c) imagining absent objects (Leslie, 1988). In pretend play, language can be used to communicate pretense. Thus, pretend play often incorporates communication (Garvey, 1982), such that conversational skills are developed within play between play partners (Sawyer, 1997).

Pretend play is closely linked to what Premack and Woodruff (1978) referred to as theory of mind – the ability to attribute mental states to others that are distinct from oneself (Leslie, 1987; Sawyer, 1997; Dunn and Cutting, 1999). Children who pretend more frequently have better theory of mind skills (see review Lillard et al., 2011). For example, Youngblade and Dunn (1995) found that the amount of pretend play predicted later abilities on a false belief task. Similarly, Schwebel et al. (1999) found that engagement in interactive pretend play positively correlated with scores on a theory of mind task measuring children’s ability to differentiate between objects’ symbolic and actual identities. The relationship between theory of mind and pretend play could be explained by specific aspects of pretend play, such as taking on the role of another, meta-representation, negotiation, or the socioemotional themes inherent in pretending (Lillard et al., 2011).

Children with autism spectrum disorder (ASD) demonstrate coreimpairment in reciprocal social communication and flexible thought and behavior (APA, 2013). Even though the diagnostic criteria have changed over time, key deficits have been constant to include impairment in social engagement and receptive and expressive language, which can include difficulty in sharing of imaginative play (Kanner, 1943; Wing and Gould, 1979; Mahjouri and Lord, 2012; APA, 2013). Children with ASD have significant difficulty socially engaging in reciprocal play with peers (e.g., Schupp et al., 2013). Some of the difficulties stem from the social challenges inherent in autism; children with ASD may demonstrate less play because they receive fewer invitations to play or experience more social failures (Jordan, 2003). Humphrey and Symes (2011) postulated a cyclical model for children with ASD, in which negative peer experiences lead to lower motivation for social interaction, thereby leading to poorer social skills (e.g., perceiving and responding to social initiations and bullying) that contribute to negative peer interactions. For example, Schupp et al. (2013) noted that, in observations of children with autism on a playground, older children with ASD demonstrated more social avoidant behavior than younger children with ASD. The authors suggested that this may be due to the history of negative social experiences that accumulate as children with autism age, in combination with increased insight into their social difficulties. In addition, core deficits in reciprocal social communication (APA, 2013) may impact the ability of children with autism to fully engage with others.

Besides deficits in social communication and engagement, individuals with ASD demonstrate restricted, repetitive, and stereotyped behavior (APA, 2013). These limitations in flexible thoughts and behaviors may result in reduced imagination and pretend play. Imagination, a central component of pretend play, is often deficient in ASD (Crespi et al., 2016). On some tests of imagination and creativity, children with autism produce less creative/imaginative responses than control groups (Craig and Baron-Cohen, 1999). Children with ASD seem to engage in more repetitive behaviors during play instead of imaginative play (Honey et al., 2007) and are less likely than typically developing (TD) children to spontaneously produce pretend play. Indeed, many studies indicate that children with ASD often exhibit deficits in pretend play (e.g., Sigman and Ungerer, 1984; Baron-Cohen, 1987; Jarrold et al., 1993). With that said, children with ASD are able to engage in pretend play when instructed to or when it is elicited (Lewis and Boucher, 1988; Jordan, 2003).

When compared to TD children, play behavior of children with autism is demonstrably different. For example, in playground observations, children with autism engaged in more Self Play (e.g., swinging on a swing), fewer interactions overall, and less cooperative play, such as playing catch (Humphrey and Symes, 2011; Schupp et al., 2013; Corbett et al., 2014b). Children with autism also tend to demonstrate more repetitive play behaviors (Honey et al., 2007) and often do not engage in pretend play (Wolfberg, 2015).

Anxiety is common among youth with ASD (White et al., 2009; van Steensel et al., 2011), and social anxiety is particularly prevalent in this population (Bellini, 2004; White et al., 2009). Accordingly, the social aspects of play seem to be more stressful and anxiety-provoking for children with ASD. For instance, Corbett et al. (2014b) measured the response of cortisol, a stress hormone, during social play on a playground in children with TD and children with ASD; while the cortisol levels in the ASD group were variable, many children with ASD showed significantly higher stress in response to social play compared to TD children. This stress may lead to socially avoidant behavior in some children with autism (see Corbett et al., 2010).

It has been speculated that deficits in social communication may contribute to social anxiety (Corbett et al., 2010; Pickard et al., 2017). Bellini (2004) found a negative correlation between adolescent self-report of social skills (i.e., Assertion subscale) and social anxiety. Furthermore, Bellini (2006b) demonstrated that social skill deficits (along with physiological arousal) were significant predictors of social anxiety in youth with autism. Again, this could be a cyclical relationship, such that poor social skills create negative peer interactions, thus leading to anxiety in social situations, subsequent avoidance, and less opportunity to improve social skills (Bellini, 2004, 2006b).

Given the importance of play in development, interventions have used a variety of approaches aimed at teaching play to children with ASD. Henning et al. (2016) described a peer- and parent-mediated intervention involving clinic visits and video modeling, which increased play in a portion of the participants; however, these gains did not generalize. Another multiple case study was conducted by Jahr et al. (2000), investigating a cooperative clinician-mediated play training intervention, which led to increased cooperative play in children with ASD. A randomized controlled trial of two play interventions targeting symbolic play (i.e., number of different novel, child-initiated symbolic play acts, from single scheme sequences to sociodramatic play) or joint attention was investigated by Kasari et al. (2006). Findings indicated improvements in each treatment group’s targeted behavior compared to a control group. Other interventions include parent-mediated behavioral interventions or facilitated peer groups (Jordan, 2003).

As some of the previously mentioned interventions demonstrate, peers can play an important role in interventions for children with autism. Corbett et al. (2014b) described how mere solicitation by peers increased the amount of social engagement in children with ASD by 30%. Not only can peers be expert role models, but they can help break the cycle of negative peer interactions leading to reduced social motivation (Humphrey and Symes, 2011). Moreover, utilizing peers can help with generalization of skills (Kamps et al., 1992).

Though play has a key role in early development, it serves as a key training ground as the skills it engenders continue to grow throughout the life span (Jordan, 2003; Goncu and Perone, 2005). Interactive play may be incorporated into different components of life and serves as a core component of theater. Play has been compared to improvisation (Sawyer, 1997) and theater more broadly (Schmitt, 1981). Goncu and Perone (2005) highlighted several similarities between play and improvisational theater, including the flexible use of symbols and context, as well as the communal nature of the activities. Given these characteristics, Schmitt (1981) posited that pretend play engaged in with others is a natural predecessor to theater.

Theater, like play, involves the use of social and imaginative skills (Kempe and Tissot, 2012; Corbett, 2016). For example, acting relies on perspective taking, and participation in theater has been shown to improve skills in theory of mind in TD youth (Goldstein and Winner, 2012) and children and adolescents with ASD (Corbett et al., 2019). Additionally, participation in theater has resulted in changes in positive interactive play in a sample of children with ASD, attention deficit hyperactivity disorder, and non-verbal learning disorder (Guli et al., 2013). Thus, theater is increasingly being used as an interventional technique. For instance, some research has implemented theater interventions to successfully develop participants’ imagination (Kempe and Tissot, 2012) and key social skills (e.g., face memory, theory of mind, and social communication with peers) (Guli et al., 2013; Corbett et al., 2016b, 2019).

As described above, many interventions aimed at improving play involve training and teaching play skills. SENSE Theatre^®^ is a novel intervention that incorporates peer mediation and theater in a supportive social atmosphere to enhance social competence in children with ASD (Corbett et al., 2014c, 2016b). Though SENSE Theatre^®^ does not explicitly teach play, it targets components vital to play. For example, engaging in imaginative play in the theater may provide a model for developing the imaginative skills necessary for pretend play and theory of mind (Goldstein and Winner, 2012). Peers are trained to implement other core objectives of SENSE Theatre^®^, including providing social support, modeling warm social interaction, and enhancing motivation (Corbett et al., 2014c). These supply positive social experiences to counter histories of negative social experiences that may lead to avoidance of social play.

In pretest/posttest and randomized clinical trial designs, participants in SENSE Theatre^®^ have demonstrated improvements in social competence, memory for faces, social functioning, theory of mind, and adaptive skills (Corbett et al., 2014c, 2016b, 2019). Notably, in a randomized clinical trial, group play at post-intervention testing was higher in the experimental (EXP) group compared to the waitlist control (WLC) (Corbett et al., 2016b). Additionally, the participants revealed less Trait anxiety at posttest in the EXP group compared to the WLC, as measured by the State-Trait Anxiety Inventory for Children (STAIC) (Spielberger et al., 1983; Corbett et al., 2016a). Correspondingly, a negative correlation was found between amount of group play and trait anxiety. While mediation analysis did not reveal a mediating role for group play, this study indicated that SENSE Theatre^®^ had positive implications for both anxiety and group play in children with ASD. However, this study was limited by the relatively small sample size. More recently, three cohorts in an extended randomized clinical trial were combined to explore changes in social competence related to SENSE Theatre^®^. Treatment effects were found on theory of mind and face recognition tasks. In addition, participants in the EXP group engaged in more reciprocal cooperative and verbal interaction behaviors than the WLC group (Corbett et al., 2019).

The current study uses the three aforementioned merged cohorts to examine anxiety and group play in ASD, specifically investigating the impact of SENSE Theatre^®^ on Group and Self Play and the anxiety that can hinder social play.

## Materials and Methods

### Participants

Three merged cohorts of participants, from three implementations of the intervention, were used for this study. A total of 102 youth, ages 8–16, were recruited through word of mouth and local support organizations. Out of the 102 youth, 87 met inclusion criteria. Of this 87, 10 participants were lost to follow-up testing. There were no significant differences on diagnostic or demographic variables (all *p* > 0.05) between participants who did and did not complete the study. Thus, the final sample consisted of 77 youth with ASD. Participants were allocated using simple randomization by a non-affiliated statistician to EXP (44 participants) and WLC (33 participants) groups. Each cohort consisted of participants who were randomized to either the EXP or WLC condition which occurred over three consecutive years [Cohort 1 in 2014 (*N* = 29), Cohort 2 in 2015 (*N* = 28), and Cohort 3 in 2016 (*N* = 20)]. Cohort 1 consisted of 17 EXP, 12 WLC (age = 10.60, 5 females, 24 males), Cohort 2 consisted of 15 EXP, 13 WLC (age = 10.99, 7 females and 21 males), and Cohort 3 consisted of 12 EXP, 8 WLC (age = 10.70, 6 females, 14 males). The EXP group received the treatment initially, and the WLC group received the treatment 6 months later. See Table 1 for demographic information; see Corbett et al. (2019) for an expanded characterization of the sample. The study is registered with www.clinicaltrials.gov ID# NCT02276534. The Vanderbilt Institutional Review Board approved this study. Informed written consent (parents) and assent (children) were obtained prior to inclusion in the study.

**TABLE 1:** Demographic and pretest variables.

Variable	EXP	WLC	*df*	χ *^2^/t*	*p*
**Demographics**					
Race					
Caucasian	36	29	1	0.53	0.47
African American	6	2	1	1.16	0.28
Asian/Pacific Islander	2	2	1	0.09	0.77
Ethnicity					
Hispanic	2	3	1	0.64	0.42
Non-Hispanic	42	30			
**Pretest variables**					
STAIC-State	31.68	31.69	1,74	0.004	1.00
STAIC-Trait	37.66	37.47	1,74	–0.10	0.92
Unsolicited (T1) Self Play	27.61	29.49	1,74	0.31	0.76
Unsolicited (T1) Group Play	8.29	7.33	1,74	–0.24	0.81
Solicited (T4) Self Play	12.42	12.78	1,74	0.06	0.95
Solicited (T4) Group Play	60.86	48.86	1,74	–1.43	0.16

*EXP, experimental group; WLC, waitlist control group; STAIC, State-Trait Anxiety Inventory for Children. The duration of play behavior is presented as seconds.*

Inclusion criteria for the study were a diagnosis of ASD and an IQ ≥ 70 using the Wechsler Abbreviated Scale of Intelligence (WASI-II; Wechsler, 2011). Participants with a history of aggression over the prior 6 months (per parent report or clinical observation) were excluded from the study for the safety of participants and staff. Diagnosis of ASD was made based on three criteria: (1) previous diagnosis by a psychologist, psychiatrist, or behavioral pediatrician with autism expertise, (2) current clinical judgment (BAC), and (3) corroboration by the Autism Diagnostic Observation Schedule-2 (ADOS 2; Lord et al., 2000) by research-reliable personnel. There were no significant differences between groups on inclusion criteria [ADOS and WASI scores, *t*(75) = −0.96, *p* = 0.32, *t*(75) = 1.00, *p* = 0.07, respectively] or on pretest variables (Table 1).

### Intervention

Ten core objectives underlie SENSE Theatre^®^: provide social support, create an enjoyable environment, model warm social interaction, enhance motivation, engage in directed communication, use non-verbal communication, engage in imaginative play, use empathic responding, support active learning, and advance individual learning (Corbett et al., 2014a).

Peers receive training on SENSE core objectives and behavioral techniques (e.g., shaping, extinction) during a full day of training; fidelity is measured by a pretest/posttest on the day of training and is tracked by trained observers during intervention sessions. These observers tracked fidelity of behavioral techniques and core objectives; booster sessions were given if fidelity fell below 80%. For behavioral techniques, mean scores across all ratings for beginning, middle, and endpoint were 91.29%, 78.75%, and 87.86%, respectively. For core principles, mean scores across all ratings at beginning, middle, and endpoint were 88.86%, 78.75%, and 89.1%, respectively. Over the years, the most significant changes to the program include enhanced and efficient training (e.g., one full day instead of 2 days of training) and rigorous implementation fidelity measures to monitor peers during intervention sessions.

For the current study, peers ranged in age from 10 to 24 years of age, but most were in high school and recruited from the University School of Nashville high school and local community theater programs. The mean age and sex per cohort were as follows: 2014 (16.74 years, 14 females, 5 males); 2015 (17.83 years, 13 females and 5 males); 2016 (17.89 years, 15 females and 3 males), indicating that they were generally comparable across the cohorts.

SENSE Theatre^®^ consisted of 10 4-h sessions. Schedules of each day’s activities were sent to families in advance and written on a whiteboard for each session. Initial sessions included mock auditions, theater games, and imaginative play. Later sessions incorporated character development, role-play, and rehearsal of the play with music. Video modeling was integrated into the program: participants practiced theater games and songs at home using videos on a secure website. The intervention culminated in two public performances.

Participants were not involved in selecting the plays. Three original theatrical plays with music were used, each lasting approximately 45 min in length and containing two group songs and one ballad. Select theater games were used that are presented in the manual and commonly used in theater (e.g., the Name Game, the Present Game) in addition to various improvisational exercises. Theater games were led by the peer actors. The participants were cast in a role commensurate with their ability regardless of age. When possible, unique talents of participants were incorporated in the show (e.g., playing an instrument).

### Dependent Measures

#### Peer Interaction Paradigm

The Peer Interaction Paradigm (PIP) is a semi-structured playground observation protocol designed to provide an ecologically valid social interaction. Two gender- and age-matched confederates assist in dividing the interaction into free and solicited play. The established paradigm is described in detail in prior work (e.g., Corbett et al., 2010; Schupp et al., 2013). Briefly, the 20-min interaction is divided into four 5-min blocks (T1, T2, T3, T4), consisting of free play (T1), solicited cooperative play (T2), free play with toys (T3), and solicited cooperative play with toys (T4). Solicited play is invited by a lead confederate and occurs during T2 and T4; unsolicited play is initiated by the participant and not invited by a confederate and occurs during T1 and T3. To ensure distinct periods of time and to contrast the measurement of behavior, only T1 (unsolicited) and T4 (solicited) were analyzed.

Confederates for the current study were TD children between 10 and 16 years of age who responded to fliers, completed an interview, and underwent protocol training. Each confederate was trained on facilitating the PIP which required him or her to play independently (T1 and T3) or solicit play from the participant (T2 and T4). The confederate was instructed to always accept the play invitations of the participant even if it occurred during independent play periods. The distinct periods of play appeared seamlessly to the participant as the confederate received cues from research personnel through a discrete earpiece with a remote transmitter. At the beginning of the paradigm, the confederate was on the playground and the timing began when the participant entered the gated play area. Therefore, the confederate provided behavioral structure to the play by permitting key interactive sequences to occur within an otherwise natural interaction and setting. The confederate for the post-visit was a child the participant had not met before, thereby representing a novel peer interaction.

As noted, the lead confederate is cued to initiate these blocks of time by communication with research personnel through an earpiece, and communication is recorded with microphones clipped to each child (Sennheiser body pack and Audio-Technica transmitters and receivers). The interaction is recorded with four professional, remotely operated cameras (70 Sony PTZ). Recorded videos were behaviorally coded using The Observer XT (Noldus, 2008).

For the current study, the time duration behaviors Group Play and Self Play on the equipment during solicited (T4) and unsolicited (T1) play were used. Specifically, the percentage of time engaged in the behavior was the unit of measurement represented in seconds. Group play was defined as the duration of activity when the participant is engaging with the group together in an activity by using the same types of equipment or toys as other members of the group. This behavior was selected based on previous research with SENSE Theatre^®^ that has shown significant treatment effects on this broad encompassing play variable (Corbett et al., 2016b). Self Play was defined as engaging in an activity independent of other children (e.g., ride a tricycle). If the child did not engage in any play activity, neither variable would be coded and it would have been coded as no play. Selected behaviors are distinct from Cooperative Play, which is reliant on reciprocal participation of two or more children (e.g., throwing a ball back and forth, playing a game). Recent findings examining cooperative play with the current cohort have been reported (Corbett et al., 2019).

#### State-Trait Anxiety Inventory for Children

The STAIC is a self-report questionnaire aimed to measure both State (current) and Trait (enduring) anxiety (Spielberger et al., 1983). It has been utilized among both TD youth (e.g., Muris et al., 1998) and youth with ASD (Lanni et al., 2012; Park et al., 2013; Simon and Corbett, 2013). Alpha reliability ranges from 0.78 to 0.91; test–retest reliability for the STAIC-Trait is 0.65–0.71 (Julian, 2011). Participants in the EXP and WLC groups were administered the STAIC after the PIP.

### Statistical Analyses

Independent sample *t*-tests were conducted to examine baseline group differences in the demographic and diagnostic variables (Table 1) and in all pretest-dependent variables. As in previous related studies, to examine between-group treatment effects, a series of linear mixed analysis of covariance (ANCOVA) models were used to test the post-intervention between-group differences on each dependent variable (Table 2). The posttest (after intervention) score for each dependent variable served as the outcome variable, group (EXP/WLC) as the main independent variable while controlling for baseline (pretest) score and Cohort (i.e., 1, 2, 3), which served as covariates. In addition, regression analyses were conducted to predict solicited Group Play based on pretreatment Group Play, age, group, and ADOS score. This was also conducted for unsolicited Group Play. Statistical analyses were performed using SPSS 24 (IBM Corp. Released 2016. IBM SPSS Statistics for Windows, Version 24.0; Armonk, NY: IBM Corp.).

**TABLE 2:** Pretest-adjusted post-mean differences for play and anxiety variables.

Variable	EXP	WLC	*df*	*F*	*p*
STAIC-State	31.48	31.28	2,71	0.07	0.93
STAIC-Trait	35.07	39.44	2,71	6.87	0.01
Unsolicited (T1) Self Play	48.83	62.64	2,73	0.46	0.50
Unsolicited (T1) Group Play	16.41	6.88	2,73	3.83	0.05
Solicited (T4) Self Play	15.43	37.09	2,73	6.70	0.01
Solicited (T4) Group Play	68.37	44.09	2,73	7.78	0.007

*EXP, experimental group; WLC, waitlist control group; STAIC, State-Trait Anxiety Inventory for Children. The duration of play behavior is presented as seconds.*

## Results

Pretest adjusted posttest findings for the dependent variables outlined below are presented in Table 2.

### Peer Interaction Paradigm

#### Solicited Play

Following treatment, children in the EXP group engaged in significantly more Group Play [*F*(2,73) = 7.78, *p* = 0.007] than children in the WLC group. Additionally, children in the EXP group engaged in significantly less Self Play [*F*(2,73) = 6.70, *p* = 0.01] than the WLC group.

#### Unsolicited Play

In regard to unsolicited activity by a peer, an increase in Group Play was marginally significant [*F*(2,73) = 3.83, *p* = 0.05]. In contrast, there was no significant difference for Self Play between the groups during unsolicited activity [*F*(2,73) = 0.46, *p* = 0.50].

#### Predictors

Regression analysis was conducted to predict solicited Group Play based on pretreatment Group Play, age, group, and ADOS score. A significant regression equation was found [*F*(4,60) = 5.943, *p* < 0.0001], with *R*^2^ = 0.284. Pretreatment Group Play (β = 0.380, *p* = 0.001) and group status (EXP/WLC) (β = 0.22, *p* = 0.05) were marginally significant predictors of solicited Group Play. A similar analysis was run for unsolicited Group Play based on pretreatment Group Play, age, group, and ADOS score. A significant regression equation was found [*F*(4,60) = 4.89, *p* = 0.002] with *R*^2^ = 0.246. Pretreatment unsolicited Group Play was a significant predictor (β = 0.434, *p* < 0.001), and group status was at trend (β = 0.216, *p* = 0.06).

### State-Trait Anxiety Inventory for Children

Children in the EXP group reported significantly less Trait anxiety than children in the WLC group following intervention [*F*(2,71) = 6.87, *p* = 0.01]; however, there was no difference in State anxiety [*F*(2,71) = 0.07, *p* = 0.935].

## Discussion

The current study corroborated previous findings on the utility of SENSE Theatre^®^ to increase Group Play and decrease anxiety in children with autism (e.g., Corbett et al., 2016a, b). Specifically, children who participated in the SENSE Theatre^®^ intervention compared to a WLC group demonstrated increased Group Play during playground interactions with novel peers. These results suggest that engaging with TD peers during the theater activities may increase motivation for children with ASD to participate in subsequent play activities. This is further supported by a decrease in Self Play in the EXP group compared to the WLC group, indicating that youth in the intervention were more likely to participate in Group, rather than Self, Play. Importantly, this observation was made during solicited play by a novel peer. During unsolicited play, there was no difference in Self Play between the EXP and WLC groups; Group Play was longer in the EXP group, but only marginally so. This underscores the significance of peer involvement in increasing engagement in play among children with ASD (Corbett et al., 2014c) and reinforces the importance of peer-mediated interventions not only for social engagement in general (Strain et al., 1979; DiSalvo and Oswald, 2002) but for play specifically (Jordan, 2003).

As is highlighted by the differences between solicited and unsolicited play, peer mediation is a crucial component of SENSE Theatre^®^. In peer-mediated interventions, peers often act as role models (DiSalvo and Oswald, 2002; Prendeville et al., 2006). Thus, in SENSE Theatre^®^, peers (as co-actors in the play) are role models for SENSE core objectives relevant to play, including non-verbal communication and imaginative play. Actors have been exposed to many of the theatrical techniques in the program and are conceptualized as expert models of reciprocal social communication and play; therefore, they are optimal trainers for the program. Peers in SENSE Theatre^®^ are trained to create a supportive environment, which may help counter negative social experiences participants have had. This supportive environment can interrupt the cycle of negative social interactions that may lead to anxiety and social avoidance (Bellini, 2006a; Humphrey and Symes, 2011).

Peers are not only role models and agents of positive social interaction but also a means to practice skills, such as social or play skills. Such practice with peers improves generalization (Kamps et al., 1992) since peers are often the target of these skills in the participant’s social environment. In this way, the context of supportive play relationships and practice in imaginative play with peers could lead to improved social play in arenas such as the school environment, as evidenced during the PIP, an ecologically valid measure of real-world social play with other children.

Along with increased play, participants in the EXP group demonstrated less anxiety – specifically, Trait anxiety as measured by the STAIC. This replicated previous findings that children with ASD who participated in the intervention self-reported reduced anxiety following social play with novel peers (Corbett, 2016).

In other words, children in the treatment group reported reduced anxiety following group play with new children who did not participate in the program and were unfamiliar to the participant. Findings that Trait, not State, anxiety was decreased in the EXP group mirror previous work (e.g., Corbett et al., 2016a). Youth with ASD may have more difficulties reporting a current affective state (Bolte et al., 2008) but seem to be able to report persistent traits, such as Trait anxiety, reliably (Simon and Corbett, 2013). In this way, the participants in the EXP group may recognize their enduring anxiety patterns as being lower yet may not identify changes in their related current feelings. Thus, the STAIC-State may not accurately reflect changes in state anxiety in youth with ASD, but the STAIC-Trait seems to be a reliable indicator of anxiety (Simon and Corbett, 2013; Corbett et al., 2016a). Since this pattern has been observed across distinct studies, it appears to be clinically meaningful.

Though the STAIC-Trait does not explicitly measure social anxiety, previous work investigated correlations of this measure, when given after various stressors, to the Multidimensional Anxiety Scale for Children (MASC) (March et al., 1997). Administered after the PIP, the STAIC-Trait was strongly related to social anxiety (Simon and Corbett, 2013). STAIC-State ratings were moderately related to the MASC social anxiety subscale but not consistently elevated in response to the given stressor (the playground) – unlike STAIC-Trait ratings. Thus, STAIC-Trait ratings could reflect some of the anxiety related to the immediate social situation (the playground).

Previous reports suggest that participation in SENSE Theatre^®^ contributes to improvement in social abilities in youth with ASD (Corbett et al., 2016a, 2019). It is plausible that this improvement (and the positive peer experiences in SENSE Theatre^®^) played a part in reducing social anxiety in the EXP group. Anxiety in social situations can lead to social avoidance (Bellini, 2006b); less anxiety can ostensibly decrease this avoidance, increasing social (Group) play. Increased play can furthermore promote additional socioemotional gains; social play itself seems to develop social competencies and skills (e.g., Connolly and Doyle, 1984; Sawyer, 1997; Lillard et al., 2011). In contrast to Humphrey and Symes (2011) who postulated a cycle in which negative peer experiences lead to lower motivation for social interaction, reduced social skills, and negative peer interactions, participation in the intervention seems to alter such a pattern. In other words, it is speculated that participation in SENSE Theatre^®^ sets into motion a positive feedback loop in which supportive social encounters with trained peers and theater activities provide an enriched environment where social skills are modeled, performed, and reinforced, leading to an increased likelihood of engaging in such behavior with others and across settings. Thus, continued gains are possible through play, ostensibly propagating a cycle of positive peer interactions which lead to increased play and social competencies.

Regression analysis revealed that pretreatment play and group status were significant predictors of posttreatment effects. This finding not only supports the impact of significant changes in the EXP group but also suggests that some fundamental level of play behavior or social motivation may be important. Children with ASD likely have sufficient basic skills, and through the theater intervention, these skills may be shaped and advanced to increase social motivation and play behavior with peers. Since there were no baseline differences between the groups for either Group or Self Play, the changes may be explained by the theater intervention.

### Limitations

Though the current study included a large sample of participants with ASD, there was a relative lack of diversity (84% Caucasian). Additionally, the participants were classified as higher functioning (IQ ≥ 70). Future research with children with co-occurring intellectual disability is needed. Follow-up work with a more diverse sample may increase the generalizability of the findings.

In addition, the study did not compare SENSE Theatre^®^ to another active control condition (ACC), thereby limiting the extent to which we can definitively state that the results are attributed to the peer-mediated, theater-based intervention. Efforts are currently underway to compare the intervention to an ACC in a large, multisite, randomized clinical trial.

### Future Directions

The current study provides a foundation for further exploration into the impact of SENSE Theatre^®^ on play and anxiety. Specifically, future work can elucidate the roles particular components of SENSE Theatre^®^ play in the demonstrated gains in play and reduced anxiety (e.g., the impact of peer mediation compared to theater games or role play). Planned future directions include investigating treatment effects for the TD peers, such as enhanced empathic responding following the intervention. Further research can additionally investigate the impact of SENSE Theatre^®^ on different types of play, such as imaginative pretend play. Finally, though the current study looked at anxiety in the context of a social situation (the PIP), social anxiety in a daily context was not specifically examined. As social anxiety can be particularly relevant to play, future work can explore changes in this construct as it manifests in the daily lives of children with ASD.

In sum, the current study extends previous research in SENSE Theatre^®^ and adds to the growing body of literature on the significant impact that theater and peer mediation may have on the core and comorbid functioning of children and adolescents with ASD. As youth with ASD have demonstrated deficits in play (Jordan, 2003) and commonly experience anxiety (White et al., 2009), the findings discussed here are clinically relevant and are a starting point for further exploration.

## Data Availability Statement

The datasets generated for this study will not be made publicly available. The data is shared with the National Database for Autism Research (NDAR). Requests to access the datasets should be addressed to the corresponding author.

## Ethics Statement

The studies involving human participants were reviewed and approved by the Vanderbilt University Institutional Review Board. Informed written consent and verbal assent was obtained from parents/guardians and children, respectively, prior to participation in the study.

## Author Contributions

The named authors made significant contributions to theinvestigation and manuscript. Specifically, SI provided the organizational and conceptual framework for the manuscript, reviewed and synthesized the relevant literature, provided interpretation of the statistical analyses, and co-wrote the initial draft of the manuscript. AK contributed to the organizational structure of the manuscript and assisted with further analysis of the findings. RM assisted with statistical analyses, reviewed relevant literature, and provided conceptual insight into the findings. MK contributed to the writing of the final manuscript. BC conceptualized the study design, ran data analysis, interpreted the findings, and co-wrote the initial draft of the manuscript. All authors read and approved the content of the work.

## Conflict of Interest

BC is the founder of SENSE Theatre^®^ but derives no financial compensation from the non-profit 501©(3) entity. The remaining authors declare that the research was conducted in the absence of any commercial or financial relationships that could be construed as a potential conflict of interest.
